# Genetic polymorphism of the extracellular region in surface associated interspersed 1.1 gene of *Plasmodium falciparum* field isolates from Thailand

**DOI:** 10.1186/s12936-021-03876-y

**Published:** 2021-08-16

**Authors:** Natpasit Chaianantakul, Tippawan Sungkapong, Jirapinya Changpad, Keawalin Thongma, Sasiwimon Sim-ut, Morakot Kaewthamasorn

**Affiliations:** 1 Department of Medical Technology, Faculty of Allied Health Sciences, Naresuan University, Phitsanulok, 65000 Thailand; 2 Veterinary Parasitology Research Unit, Department of Pathology, Faculty of Veterinary Science, Chulalongkorn University, Bangkok, 10330 Thailand

**Keywords:** Malaria, *Plasmodium falciparum*, Gene polymorphism, Variable surface antigens, SURFIN, *Surf* gene

## Abstract

**Background:**

A novel variable surface antigens (VSAs), Surface-associated interspersed proteins (SUFRINs), is a protein that is modified on the surface of infected red blood cell (iRBC). Modified proteins on the iRBC surface cause severe malaria, which can lead to death throughout the life cycle of a malaria parasite. Previous study suggested that SURFIN_1.1_ is an immunogenic membrane-associated protein which was encoded by using the *surf*_1.1_ gene expressed during the trophozoite and schizont stages. This study aimed to identify the regions of SURFIN_1.1_ and investigate the genetic diversity of the extracellular region of the *surf*_1.1_ gene.

**Methods:**

A total of 32 blood samples from falciparum malaria cases that were diagnosed in Si Sa Ket Province, Thailand were collected. *Plasmodium* genomic DNA was extracted, and the extracellular region of *surf*_1.1_ gene was amplified using the polymerase chain reaction (PCR). A sequence analysis was then performed to obtain the number of haplotypes (H), the haplotype diversity (Hd), and the segregating sites (S), while the average number of nucleotide differences between two sequences (Pi); in addition, neutrality testing, Tajima’s *D* test, Fu and Li’s D* and F* statistics was also performed.

**Results:**

From a total of 32 patient-isolated samples, 31 DNA sequences were obtained and analysed for *surf*_1.1_ gene extracellular region polymorphism. Researchers observed six distinct haplotypes in the current research area. Haplotype frequencies were 61.3%, 16.2%, and 12.9% for H1, H2, and H3, respectively. The remaining haplotype (H4-H6) frequency was 3.2% for each haplotype. Hd was 0.598 ± 0.089 with the Pi of 0.00381, and S was 15. The most common amino acid polymorphic site was E251Q; other sites included N48D, I49V, E228D, E235S, L265F, K267T, E276Q, and S288F. Fu and Li’s D* test value was − 1.24255, Fu and Li’s F* test value was − 1.10175, indicating a tendency toward negative balancing selection acting on the *surf*_1.1_ N-terminal region. The most polymorphic region was variable 2 (Var2) while cysteine-rich domain (CRD) was conserved in both the amino acid and nucleotide extracellular region of *surf*_1.1_ gene.

**Conclusions:**

The Thai *surf*_1.1_ N-terminal region was well-conserved with only a few polymorphic sites remaining. In this study, the data regarding current bearing on the polymorphism of extracellular region of *surf*_1.1_ gene were reported, which might impact the biological roles of *P. falciparum*. In addition, may possibly serve as a suitable candidate for future development of SURFIN-based vaccines regarding malaria control.

**Graphic abstract:**

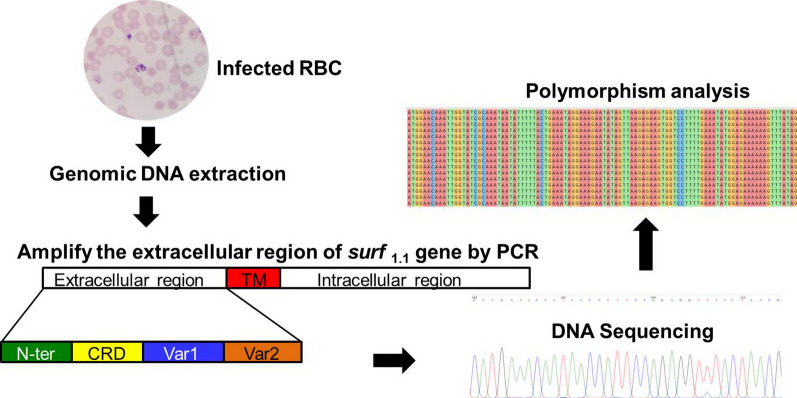

**Supplementary Information:**

The online version contains supplementary material available at 10.1186/s12936-021-03876-y.

## Highlights

- The regions of SURFIN_1.1_ were identified: SURFIN_1.1_ is comprised of extracellular, transmembrane (TM), and intracellular regions.
- Nucleotide and amino acid sequences of the extracellular region of *P. falciparum* SURFIN_1.1_ from a total of 31 field isolates were obtained and analyzed for genetic polymorphism: six different haplotypes were identified.
- The extracellular region of the SURFIN_1.1_ among field isolates was conserved, especially in the cysteine-rich domain (CRD) sub-region.
- High polymorphism was shown in the variable region 2 (Var2), followed by N-terminal (N-ter) and variable region 1 (Var1), respectively.
- The findings presented herein may enable the discovery and development of a novel SURFIN-based vaccine for prevention and control of malaria.

**Supplementary Information:**

The online version contains supplementary material available at 10.1186/s12936-021-03876-y.

## Background

Malaria caused by *Plasmodium* parasitic protozoa is one of the most serious tropical diseases faced by humans and other mammals. The disease is transmitted by the *Anopheles* spp. mosquito. There are five species of the most common strains of human malaria, including *Plasmodium falciparum*, *Plasmodium vivax*, *Plasmodium malariae*, *Plasmodium ovale,* and *Plasmodium knowlesi*. The last species (*P. knowlesi*) is capable of transmitting between macaques and humans [1, 2]. Malaria is a global public health concern. In 2018, there were 228 million malaria cases globally, with more than 4,00,000 cases of morbidity. This disease has been especially impactful amongst children under the age of five in Africa [3]. In 2016, there were 19,079 malaria cases in Thailand resulting in 33 deaths [4]. Even though malaria cases have gradually reduced globally, the mortality amongst the number of cases remains high because of the complications involved in the form of cerebral malaria [5]. In Thailand, *P. falciparum* and *P. vivax* are the most prevalent malaria species.

Cerebral malaria is the most serious form of *P. falciparum* infection, which is a consequence of sequestration (1) binding between parasite proteins on infected red blood cell (iRBC) to the endothelial cell of the microvascular system; (2) rosette formation between iRBC and normal RBC [5]. *Plasmodium falciparum* erythrocyte membrane protein-1 (PfEMP-1) is one of the major parasite-derived surface proteins located on the iRBC membrane [6]. PfEMP-1 binds to the receptors on vascular epithelial cells and becomes part of the host cell immune evasion [5, 6]. Repetitive interspersed family proteins (RIFINs) are also a binding function of iRBC to microvascular endothelial cells, which are also included in the rosette formation [7]. The PfEMP-1, RIFINs proteins, and the new parasite-derived surface protein, also known as surface-associated interspersed proteins (SURFINs), have been researched and characterized [6].

SURFINs are polymorphic proteins expressed on the iRBC membrane [6]. These proteins are encoded by using surface-associated interspersed genes (*surf* genes), which include *surf*_1.1_, *surf*_1.2_, *surf*_1.3_, *surf*_4.1_, *surf*_4.2_, *surf*_8.1_, *surf*_8.2_, *surf*_8.3_, *surf*_13.1_ and *surf*_14.1_. The *surf* genes exhibit a differential expression pattern during the different erythrocytic stages of the parasite cycle [8, 9]. The most studied and well-characterized members of SURFINs are SURFIN_4.1_ and SURFIN_4.2_, which are expressed by *surf*_4.1_ and *surf*_4.2_, respectively [9]. SURFIN_4.1_ was found in the parasitophorous vacuole (PV), but not on the iRBC membrane. SURFIN_4.2_ is accumulated in PV and found at the knobs of iRBC with PfEMP-1 [6]. Consequently, SURFIN_4.2_ might be a function in the binding of endothelial cells as well as the immune response of host cells because of the polymorphic antigen properties of this protein [6, 10].

SURFIN_4.2_ is comprised of extracellular, transmembrane (TM), and intracellular regions [6]. The extracellular region of SURFIN_4.2_ is of importance for host immune activation, antigenic variable and antigenic polymorphism on the iRBC surface [11]. Therefore, the study of SURFIN_4.2_ protein and *surf*_4.2_ gene polymorphism is designed to assist researchers in determining the benefits of potential drugs and vaccines that were developed based on research of a well-conserved region of surface antigen. There has been much research done on the SURFIN_4.2_ and the remaining nine other SURFINs. These proteins can be used to identify the expression on the surface of iRBC that might be the new target of the anti-malarial drug or new antigen for the malaria vaccine [8, 11, 12]. Among these SURFINs, SURFIN_1.1_ is one of the more interesting surface antigens that are expressed in the late stages of the parasite cycle.

A previous study showed that the *surf*_1.1_ gene expressed SURFIN_1.1_ protein during the trophozoite and schizont stages of malaria parasites [9]. The cytoadherence mechanisms of *P. falciparum* take place in these stages. Therefore, the SURFIN_1.1_ protein might function as a ligand for the binding of iRBC to vascular endothelial cells [6]. To date, no studies have examined on this protein. However, this protein is predicted to be a highly immunogenic membrane-associated protein in *P. falciparum* [13]. Therefore, the objectives of this study are to identify the regions of SURFIN_1.1_ in *P. falciparum* and investigate the extracellular region of the *surf*_1.1_ gene polymorphism in the field isolates. The findings from this study were presented in the context of the elucidation of gene polymorphism and potential targets for the development of malaria vaccines required to control the malaria disease, one of the most serious and devastating mosquito-borne diseases impacting humans.

## Methods

### Parasite isolates and DNA extraction

The research population consisted of a total 32 blood samples (isolate ID A1–A32) that were collected from symptomatic malaria-infected patients at the Kantharalak Hospital, Si Sa Ket, Thailand (2016). All clinical isolates were reported as single-species infections of *P. falciparum* as determined by light microscopic examination of Giemsa-stained blood smears. This study received ethical approval from the Naresuan University (IRB No. P10091/63).

Genomic DNA was extracted from all the isolates using a commercially available DNA extraction kit (QIAGEN, Germany) following the manufacturer’s instructions. DNA was extracted from 200 μL of whole blood in a final elution volume of 200 μL. DNA samples were kept at – 20 °C before use.

### PCR analysis of parasite species

Confirmation of the microscopic detection of *P. falciparum* and other potential co-infected species that might be in the samples were achieved using a nested PCR amplification assay based on the SSU rRNA gene [2, 14, 15].

### Identification the regions of SURFIN_1.1_

SURFIN_1.1_ (PF3D7_0113100) was analysed by comparing with previously well-studied SURFINs which included SURFIN_4.1_ (PF3D7_0402200), SURFIN_4.2_ (PF3D7_0424400), and SURFIN_1.3_ (PF3D7_0115000). These SURFINs amino acid sequences were compared with the Clustal Omega program from EMBL-EBI (www.ebi.ac.uk/Tools/msa/clustalo/). The transmembrane (TM) of SURFIN_1.1_ was also determined using a TMHMM Server v.2.0 (www.cbs.dtu.dk/services/TMHMM/).

### Amplification of the extracellular region of surf_1.1_ gene and sequencing

The extracellular region of *surf*_1.1_ was amplified with forward primer NewF (GTGCTTGTTAGAAACCCC) and reverse primer NewR (CCTTTCGAGTTGTTCCATATAC) or forward primer NewF2 (GGTGTCTTTATATACGAAAGCG) and the same reverse primer NewR. The amplification was performed in a 50 µL reaction mixture containing a 1× KOD-Plus-Neo buffer, 0.2 mM dNTPs, 1 mM MgSO_4_, 1 U of KOD-Plus-Neo DNA polymerase (Toyobo, Japan), and a 1 µL (~ 20–40 ng) of the genomic DNA template. The Thermal cycler condition includes an initial denaturation at 94 °C for 2 min; 40 cycles of 94 °C for 30 s, 55 °C for 30 s, 68 °C for 1 min; and final extension at 68 °C for 2 min.

The PCR products were analysed using a 1% agarose gel electrophoresis after ethidium bromide staining; the PCR products were then examined under UV transillumination. The PCR products were purified using a QIAquick Gel Extraction Kit (QIAGEN, Germany) and then sequencing the nucleotide sequences with the ABI 3730 DNA analyzer (Applied Biosystems) by Macrogen, Korea.

### Data analysis

The nucleotide sequences of extracellular region of *surf*_1.1_ gene were aligned using a MegAlign 15 (DNASTAR, USA). The mean numbers of synonymous substitutions per synonymous sites (*d*_S_) and non-synonymous substitutions per non-synonymous sites (*d*_N_) were computed using the Nei and Gojobori method using the Jukes and Cantor correction [16]. The statistical difference between *d*_N_ and *d*_S_ of a codon-based test was calculated with a one-tailed Z-test using 500 bootstrap replications in MEGA X [17]. A value of *d*_N_ over *d*_S_ at the 95% confidence level was considered significantly evident for positive selection. The deduced amino acids were translated from nucleotide sequences in order to investigate sequence diversity. Neutrality tests, based on measures of allele frequencies or heterozygosity within species were performed in DnaSP v6 software with the following analysis: Tajima’s *D* [18] and Fu and Li’s Tests [19]. Sliding window plots of nucleotide diversity, Tajima’s *D*, Fu and Li D* and F* tests were carried out using a 90 bp of window length and a 3 bp of step size. The secondary structure of SURFIN_1.1_ was predicted using a PSIPRED server [20, 21].

The phylogenetic analysis was constructed in MEGA X [17] by using the Maximum Likelihood method based on the Hasegawa-Kishino-Yano model [22]. The final tree was visualized in the same software with 1000 replicates Bootstrap topologies reliability test.

## Results

### The regions of SURFIN_1.1_ compared with well-known SURFINs

**Fig. 1:**
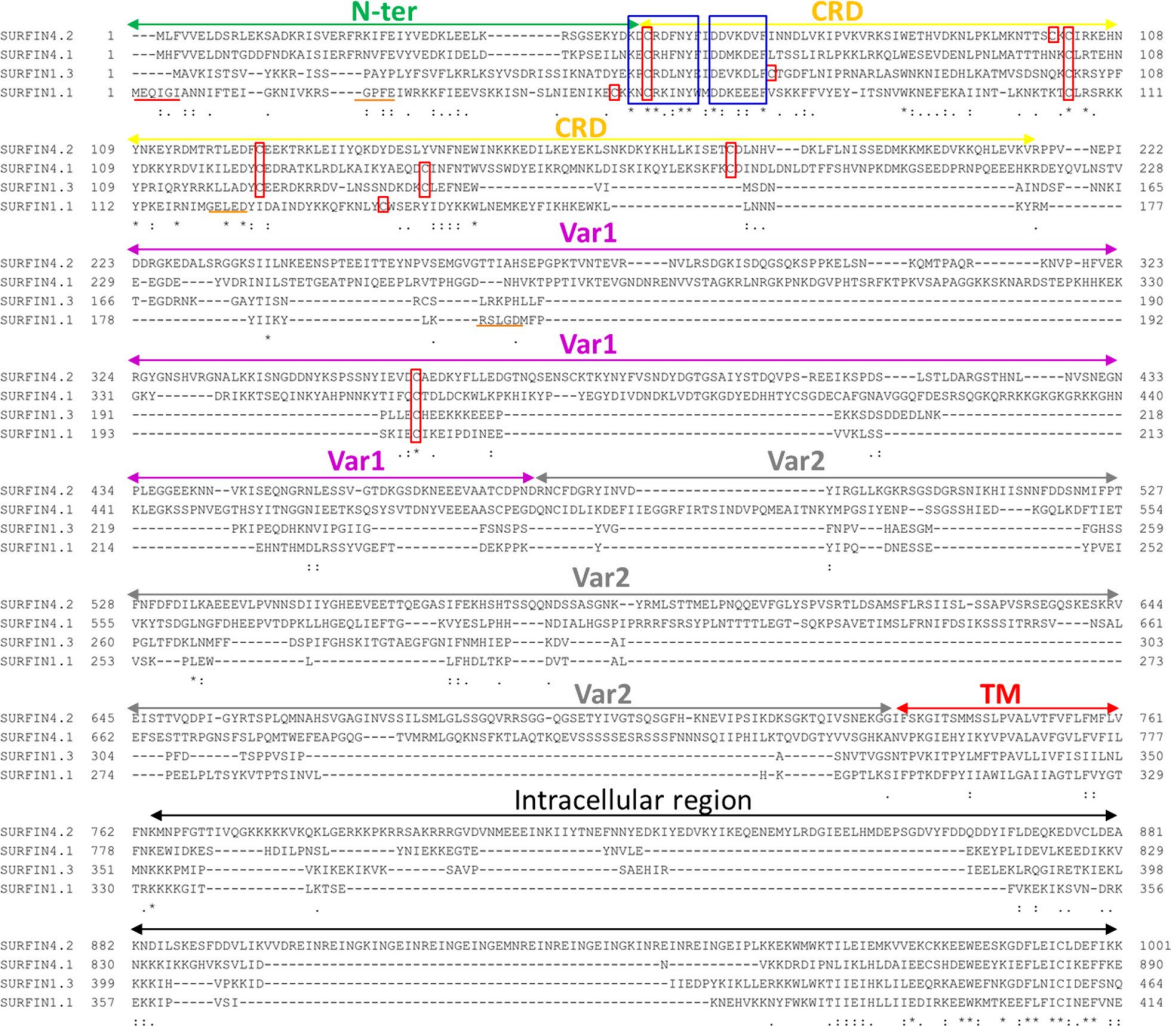
Amino acid sequences alignment of SURFIN_4.2_, SURFIN_4.1_, SURFIN_1.3_, and SURFIN_1.1_. The regions of SURFINs included extracellular (N-ter, CRD, Var1, and Var2), transmembrane (TM), and intracellular regions. Cysteine residue was marked in red box. A short conserved polar amino acid sequences were marked in blue box. The extra five amino acids in SURFIN_1.1_ were marked with red underlined. PEXEL and PEXEL-like sequences were marked with brown underlined.* = Identity amino acid, : = Highly conserved amino acid, · = Semi-conserved amino acid

**Fig. 2:**
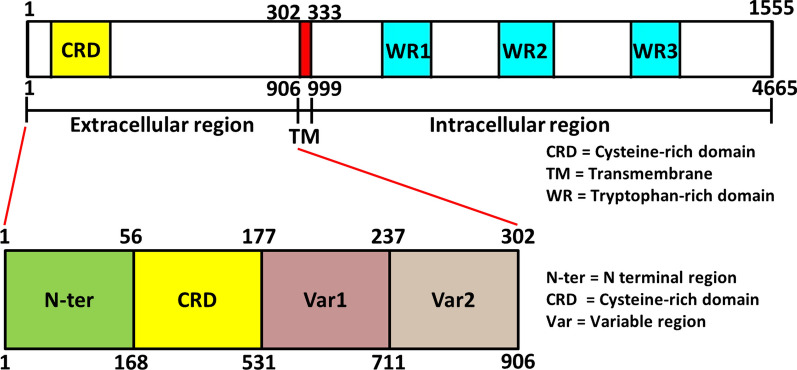
Schematic structure of SURFIN_1.1_ regions. The regions of SURFIN_1.1_ is comprised extracellular (aa 1–301), TM (aa 302–332), and intracellular region (aa 333–1555). The extracellular region of SURFIN_1.1_ was divided into four sub-regions. These sub-regions included N-ter (aa 1–55), CRD (aa 56–176), Var1 (177–236), and Var2 (aa 237–301)

To identify the regions of SURFIN_1.1_**,** the SURFINs included SURFIN_4.2_, SURFIN_4.1_, and SURFIN_1.3_, which were used as reference sequences for comparing the SURFIN_1.1_ amino acid sequences. The amino acid sequences in the extracellular, transmembrane, and some parts of the intracellular region of SURFIN_4.2_, SURFIN_4.1_, SURFIN_1.3_, and SURFIN_1.1_ were aligned (Fig. 1). The regions of each SURFIN from amino acid sequences alignment between these SURFINs were summarized, as shown in Table 1. The amino acid identity between SURFIN_1.1_ and the remaining three other SURFINs was ~ 30%. To determine the TM of SURFIN_1.1_, the amino acid sequence of SURFIN_1.1_ was analysed by using the TMHMM server. The predicted TM of SURFIN_1.1_ was located at amino acid residues 308 to 330 (Additional file 2: Fig. S1). The predicted TM amino acid position of SURFIN_1.1_ by the TMHMM server was consistent with the TM region that was analysed by using an amino acid comparison among SURFINs. The TM region of SURFIN_1.1_ is comprised of hydrophobic amino acids, which are consistent with the PfEMP1 transmembrane domain [23]. In SURFIN_4.1_ and SURFIN_4.2_, the TM region is needed for the trafficking of these proteins to the endoplasmic reticulum [24] and iRBC surface and Maurer’s cleft [25]. From these results, SURFIN_1.1_ consisting of three regions includes (1) Extracellular region: aa 1–301; (2) Transmembrane region: aa 302–332; and (3) Intracellular region: aa 333–1555 (Fig. 2). The intracellular region of SURFIN_1.1_ showed conserved tryptophan-rich domains (WR1–3) intersected by stretches of higher variability. The extracellular region of SURFIN_1.1_ was divided into four sub-regions based on the amino acid sequence of the SURFIN_4.2_ reference protein. These extracellular sub-regions of SURFIN_1.1_ included N-terminal (N-ter): aa 1–55, cysteine-rich domain (CRD): aa 56–176, variable region 1 (Var1): aa 177–236, and variable region 2 (Var2): aa 237–301 (Fig. 2).

**Table 1:** The regions of SURFINs and amino acid position

SURFIN	Length (amino acid)	MW (kDa)	Extracellular region				Transmembrane region (TM)	Intracellular region	Identity to SURFIN_1.1_ (%)
			N-ter	CRD	Var1	Var2			
4.2	2380	286	1–50	51–195	196–482	483–733	734–764	765–2380	30.2
4.1	2156	258	1–50	51–198	199–492	493–749	750–780	781–2156	29.1
1.3	1925	235	1–50	51–155	156–241	242–322	323–353	354–1925	31
1.1	1555	192	1–55	56–176	177–236	237–301	302–332	333–1555	–

*N-ter* N-terminal region, *CRD* Cysteine-rich domain, *Var1* Variable region 1, *Var2* Variable region 2

### Nucleotide polymorphism of the extracellular region of the surf_1.1_ gene

**Fig. 3:**
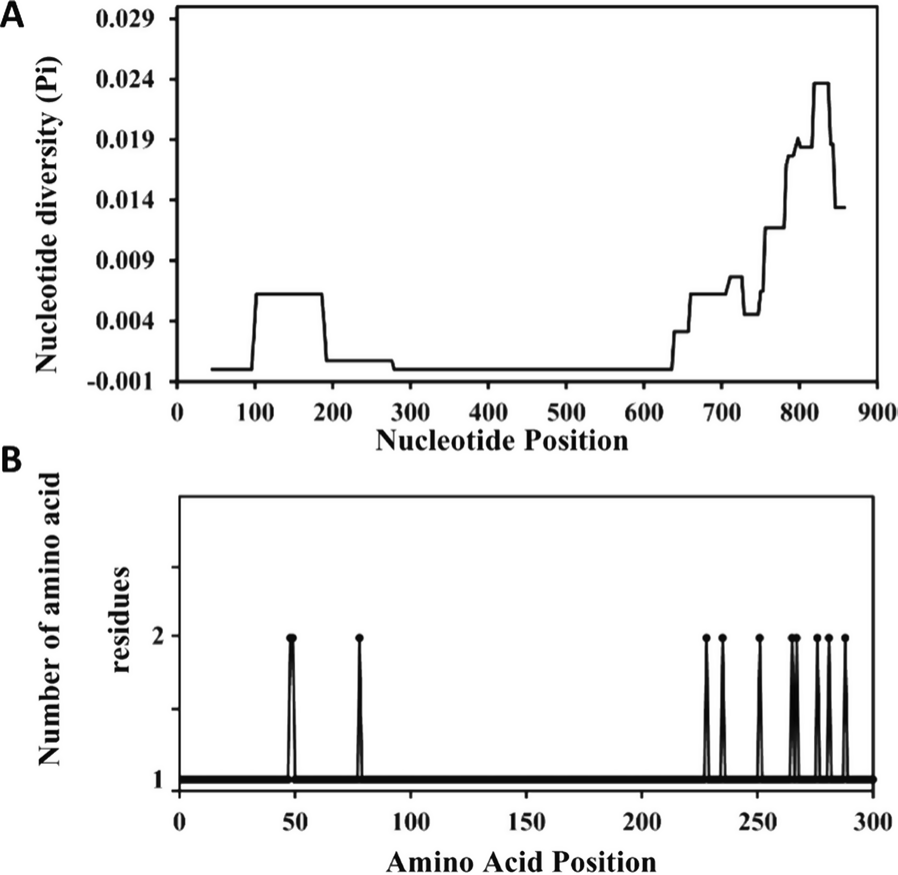
Sliding window plot of nucleotide diversity (Pi) and amino acid polymorphism of SURFIN_1.1_ extracellular region in *P. falciparum* Thai isolates. Nucleotide diversity is plotted with a window length of 90 bp and step size of 3 bp (**A**) and the number of the amino acid residues at each amino acid position is plotted (**B**). A total of 31 sequences from Thai isolates were used. Nucleotide and amino acid positions are after the 3D7 line sequences

From a total of 32 patient-isolated samples, 31 DNA sequences (excepted for isolate A18) were obtained and analysed for *surf*_1.1_ gene extracellular region polymorphism. The nucleotide polymorphism among the 31 patients isolated samples and *P. falciparum* 3D7 reference strain showed nucleotide identity at 99–100% with divergence of 0–1% (Additional file 1: Table S1). This result indicated that the extracellular region among field isolates *P. falciparum* was conserved. The nucleotide sequence compared with the *P. falciparum* 3D7 reference strain showed six different haplotypes (H1–H6). Haplotype frequencies were 61.3%, 16.2%, and 12.9% for H1, H2, and H3, respectively (Table 2). The remaining haplotype (H4–H6) frequency was 3.2% for each haplotype. The haplotype (gene) diversity (Hd) was 0.598 ± 0.089 with a nucleotide diversity (Pi) of 0.00381 (Table 3). The average number of nucleotide differences (k) was 3.441. A sliding window plot of nucleotide diversity indicates an elevation in Pi values between nucleotide positions 55 to 321 and 595 to 903 (Fig. 3).

**Table 2:** *Surf*_1.1_ nucleotide and amino acid mutation patterns of six different haplotypes identified from Thai isolates

Haplotype	Nucleotide mutation	Amino acid mutation	Number of isolates	Frequency (%)
Haplotype 1	A142G A145G G751C T756C G795T A800C G826C	N48D I49V E251Q I252* L265F K267T E276Q	19	61.3
Haplotype 2	A142G A145G G751C T756C C863T	N48D I49V E251Q I252* S288F	5	16.2
Haplotype 3	A684C C703T G751C T756C C863T	E228D P235S E251Q I252* S288F	4	12.9
Haplotype 4	T232G A684C C703T G751C T756C C863T	Y78D E228D P235S E251Q I252* S288F	1	3.2
Haplotype 5	A142G A145G G795T A800C C863T	N48D I49V L265F K267T S288F	1	3.2
Haplotype 6	A142G A145G G751C T756C G795T G826C A831G G840A G842A C843T	N48D I49V E251Q I252* L265F E276Q L277* T280* T280* S281N	1	3.2

^*^Silence mutation

**Table 3:** Sequence analysis of *P. falciparum surf*_1.1_ extracellular region from Thai isolates

Analysis	Result	
Number of Haplotypes (H)	6	
Haplotype (gene) diversity (Hd)	0.598	± 0.089
Nucleotide diversity (Pi)	0.00381	
Variance of haplotype diversity	0.00789	
Average number of pairwise nucleotide differences (k)	3.441	
Fu and Li’s D* test	− 1.24255	*P* > 0.10
Fu and Li’s F* test	− 1.10175	*P* > 0.10
Tajima’s *D* test	− 0.27968	*P* > 0.10
Number of segregating sites (S)	15	
Number of non-synonymous sites (pN) Tajima’s D (NonSyn)	11 0.49830	*P* > 0.10
Number of synonymous sites (pS) Tajima’s D (Syn)	4 − 1.88923	*P* < 0.05
pN/pS ratio Tajima’s D (NonSyn/Syn) ratio	2.75 − 0.26376

**Fig. 4:**
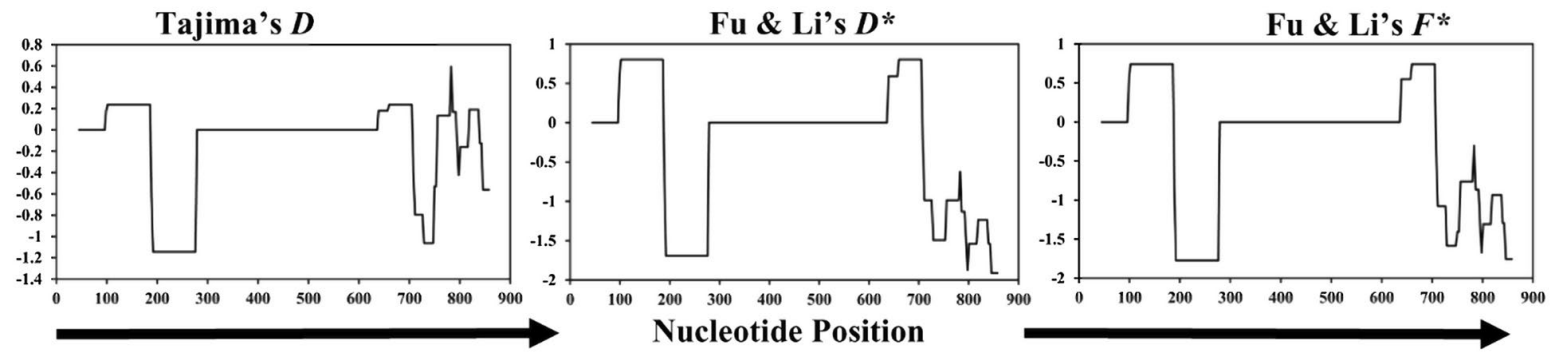
Sliding window plots of Tajima's test (*D*), and Fu and Li's tests (D* and F*) for *P. falciparum surf*_1.1_ sequence encoding extracellular region in Thai isolates. Window length is 90 bp, and step size is 3 bp

A statistical analysis of nucleotide sequences is concluded in Table 3. For the neutrality tests of the extracellular region of *surf*_1.1_, Tajima’s *D* test, Fu and Li’s D* and F* statistics were performed. Fu and Li’s D* test statistics value was − 1.24255 (*P* > 0.10), Fu and Li’s F* test statistics value was − 1.10175 (*P* > 0.10). Sliding window plots of Tajima’s *D*, Fu and Li’s D* and F* statistics show a similar tendency of the test values heading in the direction of negative selection at nucleotide positions 145–321 and 664 towards the C-terminal side (Fig. 4). It is notable that, in the sliding window plots of Tajima’s *D*, Fu and Li’s D* and F* tests, positive values were observed at nucleotides between position 100 to 200 and 650 to 700. However, statistical tests did not detect any significant value in each window. The number of segregating sites (S) detected in the extracellular region of *surf*_1.1_ was 15, and no insertion or deletions were observed in the overall samples sequenced. Tajima’s *D* value was − 0.27968 (*P *> 0.10).

**Fig. 5:**
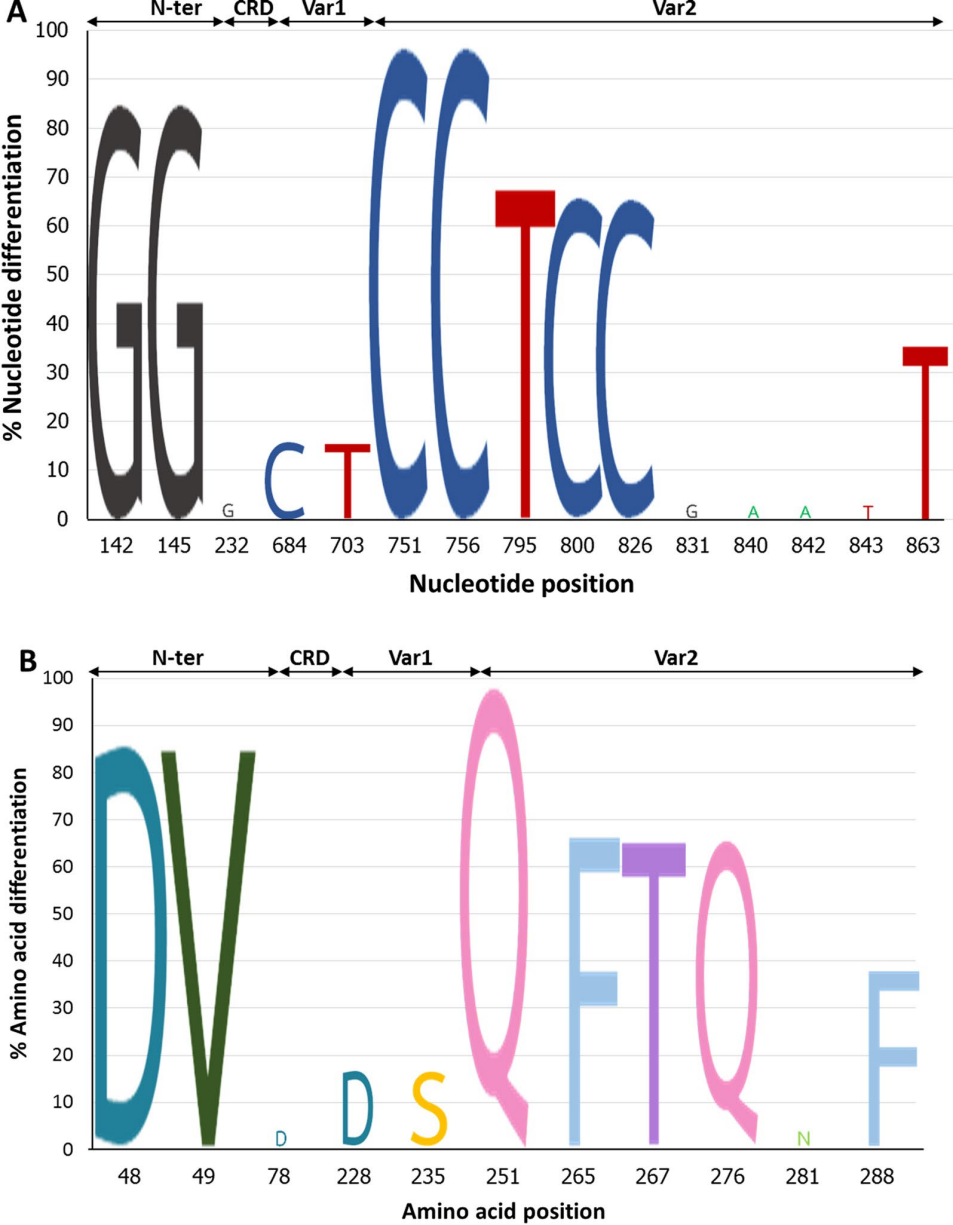
Nucleotide polymorphism (**A**) and amino acid polymorphism (**B**) in the extracellular sub-regions of *surf*_1.1_ included N-ter, CRD, Var1, and Var2 compared to *P. falciparum* 3D7 reference strain. The most polymorphic region was Var2 while CRD was conserved in both the nucleotide and amino acid sequence

The extracellular region of the *surf*_1.1_ gene was divided into four sub-regions based on amino acid sequence conservation among SURFIN proteins. The extracellular sub-regions of *surf*_1.1_ included N-ter (nt 1–168), CRD (nt 169–531), Var1 (nt 532–711), and Var2 (nt 712–906). Although the polymorphic sites were distributed across the entire extracellular sub-regions nucleotide sequence, high polymorphism was found in Var2, followed by N-ter and Var1 (Fig. 5A). The most common nucleotide polymorphisms were G751C and T756C (silence mutation) in Var2. Among six different haplotypes, H6 was a highly polymorphic isolate with 10 nucleotide mutation positions. These mutations included 4 silence mutations: T756C, A831G, G840A, and G842A. These silence mutations were observed in Var2. Interestingly, no nucleotide mutation in CRD was observed in H1, H2, H3, H5, and H6. However, there was a T232G mutation in H4.

### Amino acid polymorphism of the extracellular region of SURFIN_1.1_ protein

The 31 nucleotide sequences were translated to amino acid sequences and analysed for SURFIN_1.1_ amino acid polymorphism. The amino acid polymorphism amongst 31 patient-isolated samples and *P. falciparum* 3D7 reference strain showed amino acid identity at 97–100% with divergence of 0–3.1% (Additional file 1: Table S2). These results indicated low genetic diversity in the extracellular region of SURFIN_1.1_. The extracellular sub-regions of SURFIN_1.1_ included N-ter (aa 1–55), CRD (aa 56–176), Var1 (aa 177–236), and Var2 (aa 237–301). Few amino acid polymorphisms have been identified amongst these sub-regions. Amino acid mutations included N48D, I49V, Y78D, E228D, E235S, E251Q, L265F, K267T, E276Q, S281N, and S288F (Table 2). The most common amino acid mutation was E251Q. There were six different haplotypes, with the most frequent haplotype being H1 (19 isolates, 61.3%). The amino acid mutations in H1 included N48D, I49V, E251Q, L265F, K267T, and E276Q.

**Fig. 6:**
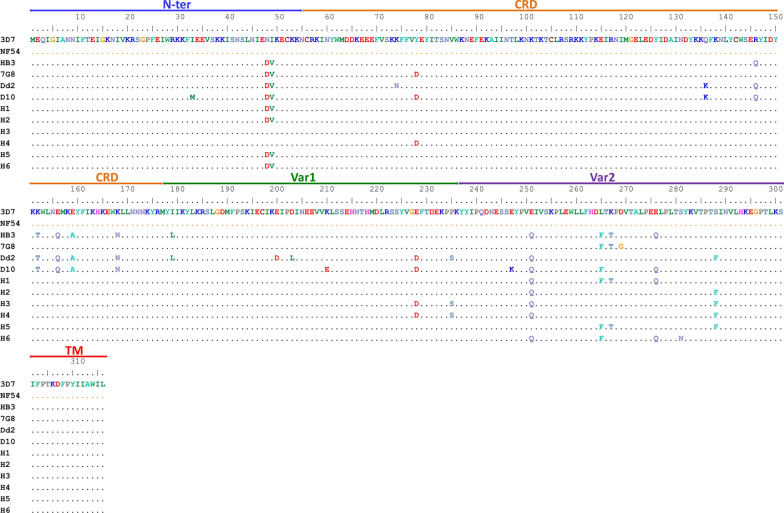
Amino acid sequences alignment of the extracellular region of SURFIN_1.1_ from six different haplotypes (H1–H6) compared to the global reference strains. The identity and mutation amino acid residues were shown in each region

Among 11 amino acid mutation positions, 2 mutations were observed in N-ter (aa position 48 and 49), 1 mutation was observed in CRD (aa position 78), 2 mutations were observed in Var1 (aa position 228 and 235), and 6 mutations were observed in Var2 (aa position 251, 265, 267, 276, 281, and 288) (Fig. 5B). Therefore, more amino acid polymorphism accumulated towards the C-terminal of the extracellular region of SURFIN_1.1_. The amount of polymorphic amino acid sites per length of amino acids in N-ter (2/55 = 3.6%) and Var1 (2/60 = 3.3%) was comparable, whereas a large number of polymorphic sites were observed in Var2 (6/65 = 9.2%) with six missense mutations in this sub-region. Interestingly, there was no amino acid mutation in CRD amongst five haplotypes except for H4 (Fig. 5B, 6, Additional file 3: Fig. S2). In H4, there was a Y78D mutation (Table 2) in CRD, which was found in only 1 isolate (3.2%). The N-ter of SURFIN_1.1_ in patients isolated showed N48D and I49V mutations, which were different from the *P. falciparum* 3D7 reference strain. These results indicated that the CRD was a highly conserved region among these field isolate parasites. The Var2 amino acid residues amongst field isolates showed consensus amino acid residues, including 251Q, 265F, 267T, and 276Q (Fig. 5B, Additional file 3: Fig. S2).

### Sequence analysis of the extracellular region of SURFIN_1.1_from different haplotypes compared with reference strains

The amino acid sequences of the extracellular region from six different haplotypes were compared to global reference strains including 3D7, NF54, 7G8 (Brazil), HB3 (Honduras), Dd2 (Indochina), and D10 (Papua New Guinea) (Fig. 6). In N-ter, N48D and I49V mutations were observed in H1, H2, H5, and H6 (26/31 isolates = 83.9%). These amino acid mutations have been reported in the HB3, 7G8, D10, and Dd2 reference strains. Therefore, these mutations might be beneficial for the field *P. falciparum* parasite. However, the molecular function of these amino acid residues in SURFIN_1.1_ must be clarified.

In the conserved CRD region, there was Y78D mutation that was found only in H4 (1/31 isolate = 3.2%). Interestingly, this mutation has been reported in 7G8 and D10 strains. This region was highly conserved among Thai isolates, which were observed in H1, H2, H3, H5, H6 (30/31 isolates = 96.8%). However, other amino acid mutations in CRD have been reported in HB3, Dd2, and D10 strains. In Var1, there were E228D and P235S mutations in H3 and H4 (5/31 isolates = 16.1%). These mutations in Var1 have been reported in Dd2 and D10 strains. For the highly polymorphic region Var2, there were E251Q, L265F, and E276Q mutations in H1 and H6 (20/31 isolates = 64.5%). These mutations in Var2 have been reported in HB3 and D10 strains.

The phylogenetic tree was constructed to describe nucleotide substitution with the reference strains. The results showed that most of the Thai *surf*_1.1_ extracellular region sequences including H1 (19 isolates) and H6 (1 isolate) were not related to those reference strains (Additional file 4: Fig. S3). However, H3 (4 isolates), H4 (1 isolate), and H2 (5 isolates) were related to 3D7 and NF54 reference strains. Interestingly, H5 (1 isolate) was firmly related to the 7G8 strain.

### Structural characteristics of SURFIN_1.1_ peptide chains

**Fig. 7:**
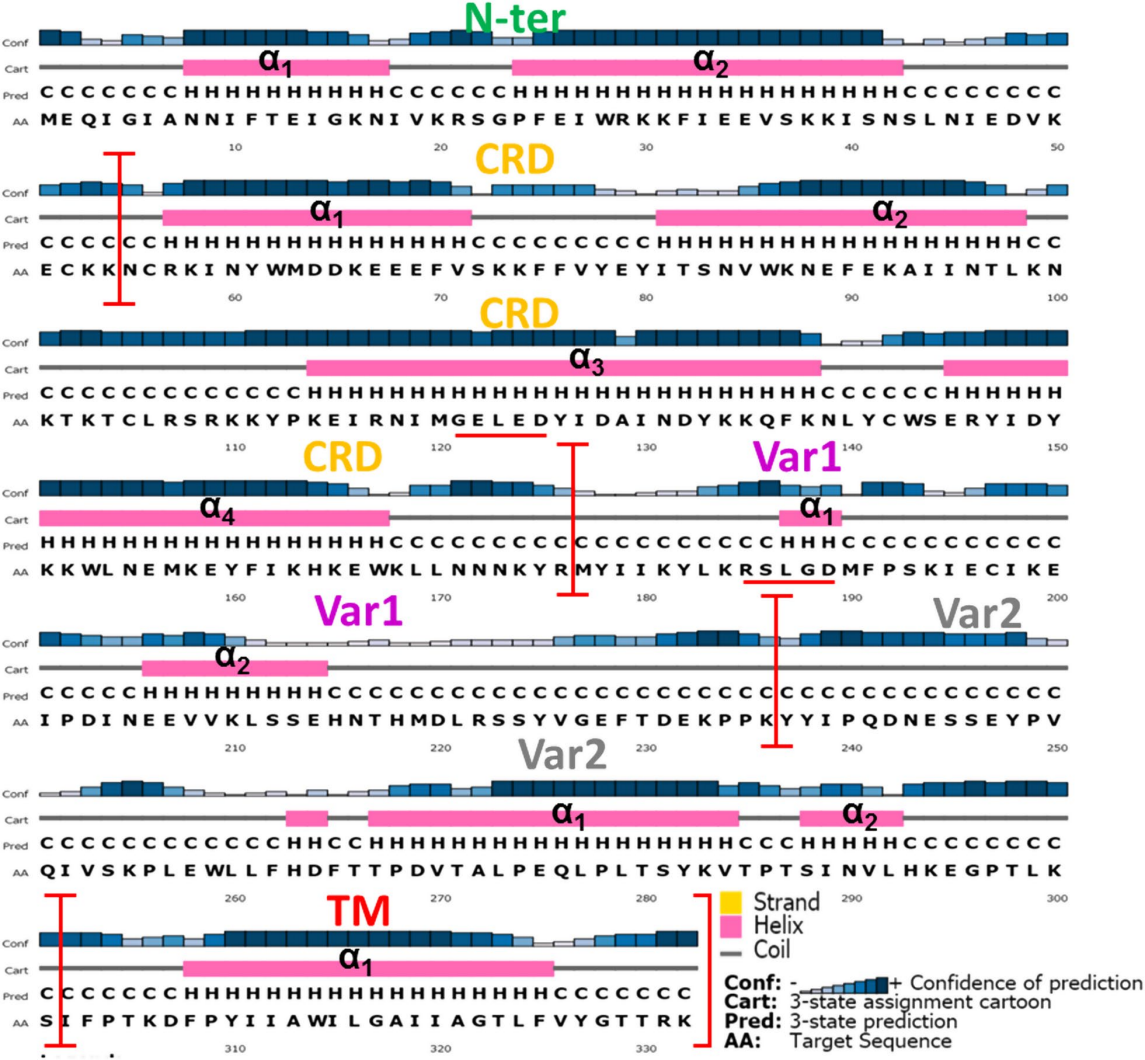
The predicted secondary structure of SURFIN_1.1_ extracellular and TM region. The amino acid sequence of the high-frequency haplotype (H1) was used for the prediction using a PSIPRED server. The PEXEL and PEXEL-like sequences were marked with red underlined

SURFIN_1.1_, together with SURFIN_4.2_ and SURFIN_4.1_, are members of the SURFIN multi-gene family. The identity between SURFIN_1.1_ to SURFIN_4.2_ and SURFIN_4.1_ was 30.2% and 29.1%, respectively (Table 1). Amino acid sequence alignment of the extracellular region from the field isolated samples showed a unique amino acid sequence. In the SURFIN_1.1_ amino acid sequence, there was a *Plasmodium* export element (PEXEL) sequence (R_185S_L_187G_D_189_) that was located in Var1. The PEXEL-like sequence (G_121E_L_123E_D_125_) was also observed in CRD (Fig. 7, Additional file 3: Fig. S2). In the N-ter of SURFIN_1.1_, there were amino acid residues 16-20 (K_16N_I_18V_K_20_). Interestingly, the K_16_, I_18_, and K_20_ residues were the same categories with amino acid residues of the PEXEL motif sequence (RxLxE/Q/D).

The amino acid sequence of the high-frequency haplotype (H1) was used for the prediction of SURFIN_1.1_ extracellular and TM region secondary structure. The N-ter of the SURFIN_1.1_ extracellular region showed five extra amino acid residues (MEQIGI) compared to other SURFINs. These amino acid residues were conserved amongst 31 field isolated SURFIN_1.1_ (Additional file 3: Fig. S2). The predicted secondary structure of H1 SURFIN_1.1_ showed a coil structure in the region (Fig. 7). In the conserved CRD region, most field isolates of SURFIN_1.1_ comprised four domains of alpha-helix (α_1-4_), which might be important for the trafficking of SURFIN_1.1_. Interestingly, in the 3^rd^ helix (α_3_) of CRD, PEXEL-like sequence (G_121E_L_123E_D_125_) was shown in this region. The PEXEL-like sequence has been shown to be responsible for the trafficking of SURFIN_4.2_ [25]. In the Var1 region, there was a short helix of PEXEL sequence (R_185S_L_187G_D_189_) that is necessary for *P. falciparum*-derived proteins exported onto the iRBC surface [26].

## Discussion

From previous studies, SURFINs showed structural and sequence similarity with exported iRBC surface proteins including PvSTP1, PkSICAvar, PvVIR, Pf332, and PfEMP1 [6]. However, the presence of SURFINs is different from other iRBC surface proteins. SURFIN_4.1_ is present within PV, around free merozoites as merozoite-associated material, but is not found on the iRBC surface [9]. For SURFIN_4.2_, it not only accumulated in the PV, but was also expressed and present in the apex of merozoite [6]. From these studies, the indication is that these SURFINs may be important to malaria parasites because the antibody to SURFIN_4.2_ was found to inhibit merozoite invasion and rosetting formation [6, 11]. Therefore, the function of SURFIN_1.1_ may involve merozoite invasion as the SURFIN_4.2_ [27]. However, the function and molecular basis of SURFIN_1.1_ need to be further clarified.

The regions of SURFIN_1.1_ were identified by comparing with SURFIN_4.2_, SURFIN_4.1_, and SURFIN_1.3_. The extracellular sub-regions of SURFIN_1.1_ include N-ter, CRD, Var1, and Var2. Interestingly, the N-ter of SURFIN_1.1_ contains a stretch of five unique MEQIGI amino acid residues that are missing in other SURFINs. In a previous study, the N-ter of SURFIN_4.1_ was needed for protein translocation across the PV of the parasite [24]. Therefore, these five amino acid residues might be important for SURFIN_1.1_ trafficking. However, further study should be carried out on this stretch of five unique amino acids to verify this hypothesis. The CRD of SURFIN_1.1_ contains four cysteine residues that have been reported in SURFIN_4.1_ [9, 24] and SURFIN_4.2_ [6, 28]. However, the molecular function of these cysteine residues has not been elucidated. Amino acid sequence alignment among SURFIN_4.2_, SURFIN_4.1_, SURFIN_1.3_, and SURFIN_1.1_ revealed a short conserved sequence of positively charged and polar amino acids (K_X_CR_XX_NY) as well as negatively charged amino acids (DD/E_XX_D/E) between the N-ter and CRD region (Fig. 1.). These results were consistent with a previous report that showed the highly-negative charged residues in the N-terminal are required for transportation of PfSBP1 into the iRBC [29]. Therefore, these amino acid residues might be important for the trafficking of SURFIN proteins during the erythrocytic life cycle. The PEXEL motif has been reported for parasite-derived protein trafficking in previous studies [25, 30]. However, the possibilities of these amino acid residues need to be evaluated in the future.

The intracellular region of SURFIN_1.1_ was comprised of conserved WR domains, which were consistent with SURFIN_4.1_ [24], SURFIN_4.2_ [25], and *P. vivax* transmembrane protein: PvSTP1 [6, 31]. The previous report shows that the cytoplasmic region, WR1 of SURFIN_4.2_ is needed for protein trafficking from Maurer’s cleft to the iRBC membrane utilizing of co-transportation with PfEMP1 and RIFIN to the iRBC surface [28]. The function of WR domains in SURFIN_4.2_ has been demonstrated, the WR2 of SURFIN_4.2_ bound to F-actin and spectrin of RBC membrane [27]. Therefore, these WR regions might be important for the function of SURFINs during merozoite invasion to normal RBC and/or rosette formation.

Nucleotide polymorphism of the extracellular region of the *surf*_1.1_ gene compared with *P. falciparum* 3D7 reference strain showed six different haplotypes. Among these haplotypes, H1 was the most high-frequency haplotype (19 isolates, 61.3%). The extracellular region of the *surf*_1.1_ gene among field isolates was conserved, especially in the CRD sub-region. High polymorphism was shown in Var2, followed by N-ter and Var1, respectively. This conservation is also observed in its analogous gene *surf*_4.2_ [10] and *Pfcsp* gene [32]; these genes showed low genetic diversity in the N-ter, CRD, and Var1. Fu and Li’s F* and D* test statistic and Tajima’s *D* test values, which indicated a probable role in negative balancing selection [18, 19, 32] occurring toward the C-terminal side of the *surf*_1.1_ extracellular region. A significant excess of non-synonymous substitutions (pN) over synonymous substitutions (pS) was detected when the entire sequence of *surf*_1.1_ extracellular region was evaluated (*p* < 0.05). The pN/pS ratio was observed at 2.75, indicating the possible role of positive selection on the extracellular region of the *surf*_1.1_ gene. This finding was consistent with a previous report that suggested an entire sequence of *surf*_1.1_ gene underwent diversifying selection with a pN/pS ratio of 4.33 [13].

Amino acid polymorphism of the extracellular region of SURFIN_1.1_ protein among 31 field isolates showed 11 amino acid polymorphic sites in four sub-regions including N-ter, CRD, Var1, and Var2. Among these regions, the most polymorphic site was the Var2 region. This result was consistent with a previous report in SURFIN_4.2_ that showed Var2 was the most highly polymorphic region [10]. Interestingly, N-ter of SURFIN_1.1_ in the field isolate parasites showed consensus amino acid residues including 48D and 49V. These amino acid residues were different from the *P. falciparum* 3D7 reference strain. These results indicated that the N-ter of SURFIN_1.1_ might be under strong diversifying selection as apical membrane antigen 1 [33]. Relatively low amino acid polymorphism in CRD of SURFIN_1.1_ supports the concept that this region could be an ideal module for a SURFIN_1.1_-based vaccine. This concept has been reported in the RTS,S malaria vaccine. The development of a regional vaccine based on the conserved region of circumsporozoite protein (PfCSP) was proposed [32]. A consensus amino acid residues in Var2 included 251Q, 265F, 267T, and 276Q. These amino acid residues in Var2 from the field isolate parasites were different from the *P. falciparum* 3D7 reference strain. Therefore, these amino acid residues might have a positive selection for the field *P. falciparum* to overcome the host immune response. However, the molecular function of these amino acid residues in SURFIN_1.1_ must be elucidated further.

The amino acid sequences of identified haplotypes H1-H6 were compared with different reference strains including 3D7, NF54, 7G8, HB3, Dd2, and D10. The N48D and I49V mutations in N-ter of SURFIN_1.1_ (found in 26 Thai isolates) have been reported in HB3, 7G8, D10 and Dd2 strains. Therefore, this region of SURFIN_1.1_ could be considered when designing a universal SURFIN_1.1_-based vaccine. Interestingly, the most common E251Q mutation in Var2 (found in 30 Thai isolates) has been reported in HB3, Dd2, and D10 strains. In a previous report, glutamate and lysine residues in N-ter were shown to be important for the export of SURFIN_4.2_ to the iRBC [34]. Therefore, these amino acid mutations in the field *P. falciparum* might be important for the trafficking of SURFIN_1.1_. However, the molecular function of these amino acid residues in SURFIN_1.1_ must be clarified. Amino acid sequence alignment between haplotypes identified from this study and reference strains showed a conserved hydrophobic TM region (Fig. 6) at amino acid positions 302-316. This result was consistent with amino acid sequence alignment among SURFINs (Fig. 1) and a predicted TM by using TMHMM servers (Additional file 2: Fig. S1). From the phylogenetic analysis, the Thai isolates haplotype 1 and 6 (20 isolates) were not related to the reference strains. Therefore, these mutation patterns observed in the SURFIN_1.1_ extracellular region from the field *P. falciparum* might be beneficial for the intra-erythrocytic development of parasites [35].

Most parasite-derived antigens on the surface of iRBC contain a pentameric amino acids sequence (R_X_L_X_E/Q/D) called PEXEL [36, 37] or vacuolar translocation signal/VTS [26, 38]. These specific amino acid sequences are necessary for the molecular trafficking of parasite-derived proteins to the surface of iRBC. However, the trafficking of SURFIN to the iRBC surface is PEXEL-independent but needs a specific region for transport across PV, Maurer’s clefts, and ER [24, 25, 28]. The PEXEL-like sequence was proposed in the molecular trafficking of SURFIN_4.2_ to the iRBC and Maurer’s clefts. In SURFIN_4.2_, the PEXEL-like sequence includes amino acid residues R_25K_I_27F_E_29_, and the PEXEL sequence includes amino acid residues R_118T_L_120E_D_122_. Even though these amino acid sequences were not observed in the N-ter of SURFIN_1.1_, there were amino acid residues K_16N_I_18V_K_20_ in the N-ter. These amino acid residues were the same categories as the amino acid residues of the PEXEL sequence. Therefore, these amino acid residues in N-ter might be important for the molecular trafficking of SURFIN_1.1_. However, further studies must be engaged to dissect the functional roles of this amino acid sequence in SURFIN_1.1_.

SURFIN_1.1_ is one of the more highly immunogenic antigens among other SURFINs [13]. Therefore, SURFIN_1.1_ might be a candidate for vaccine development because of its highly conserved variant surface antigens on iRBC. A SURFIN_1.1_-based vaccine could overcome the antigenic diversity of parasites. The conserved regions include CRD, N-ter, and Var1, which might be beneficial for a conserved epitope vaccine development [8] that could inhibit merozoite invasion or rosette formation. The antigenic diversity of parasite-derived proteins is one of the major challenges for current vaccine candidate development. The diversity of PfEMP1 and PfCSP has been reported and resulted in the escape of parasites from the host immunity [8, 32]. However, whether SURFIN_1.1_ protein potentially elicits humoral immunity or mediates immune evasion remains to be investigated.

From the predicted secondary structure of SURFIN_1.1_, amino acid residues MEQIGI formed a coil structure (Fig. 7). In a previous report, the coiled structure promotes peptide penetration through the cell membrane [39, 40]. Therefore, this conserved amino acid sequence might be important for the molecular function of SURFIN_1.1_. This design might be necessary for the trafficking of SURFIN_1.1_ to iRBC and/or invasion of merozoite to RBC. From a previous study, the PEXEL cleavage and N-acetylated (Ac-xE/Q/D) contains glutamate and glutamine residues; it is recognized by the putative translocon at the PVM [30, 37]. In a previous report, glutamate residue was also found to be critical for the trafficking of REX2 [41, 42]. Therefore, glutamate and glutamine residues observed in N-ter might be important for SURFIN_1.1_. The structural characteristics of SURFIN_1.1_, the PEXEL-like and PEXEL sequences were observed in CRD (G_121E_L_123E_D_125_) and Var1 (R_185S_L_187G_D_189_), respectively. These amino acid residues might be important for SURFIN_1.1_ function and the trafficking of protein to iRBC. These amino acid residues might interact with a putative translocon protein complex that has been reported for protein trafficking [43]. However, this hypothesis must be elucidated by using the mutagenesis analysis of these amino acid residues [44, 45]. The mutation of these amino acid residues might affect the transport of SURFIN_1.1_ and altered parasite-derived surface proteins on iRBC. The predicted secondary structure of TM (Fig. 7) showed a helix structure in this region at amino acid positions 308-325. This result was consistent with SURFINs amino acid sequence alignment (Fig. 1) and TM prediction using TMHMM servers (Fig. S1).

Currently, there are anti-malarial resistant parasites because of the antigenic variation of VSAs and the amino acid mutation of targeted-malarial enzymes. Therefore, developing an effective vaccine and anti-malarial drug is a challenge. The genetic diversity of SURFIN_1.1_ in the Thai isolates presented in this study offers a new conserved surface protein on iRBC that could be a potential option for malaria vaccine development.

## Conclusion

In the present work, the regions of SURFIN_1.1_ were identified. The SURFIN_1.1_ is comprised of the extracellular, TM, and intracellular regions. The extracellular region of SURFIN_1.1_ from the Thai isolates was conserved, especially in the N-ter and CRD region. These results suggested that this surface protein might be essential for *P. falciparum* during the erythrocytic life cycle of a parasite. However, further investigations are needed to determine its biological function during the *P. falciparum* developmental stages. Through understanding this gene and protein polymorphism, it may be possible to identify the geographical distribution, changing patterns, and immunity function of this surface protein. The findings presented herein may enable the discovery and development of a novel SURFIN-based vaccine for the global prevention and control of malaria.

## Supplementary Information

**Additional file 1: Table S1.** Nucleotide identity among 31 field isolates (excepted for isolate A18) and P. falciparum 3D7 reference strain. **Table S2.** Amino acid identity among 31 field isolates (excepted for isolate A18) and P. falciparum 3D7 reference strain.

**Additional file2: Figure S1.** The predicted transmembrane region (TM) of SURFIN1.1. The TM was predicted by using TMHMM servers. The predicted TM of SURFIN1.1 was located at amino acid residues 308 to 330.

**Additional file3: Figure S2.** Amino acid sequences alignment of the extracellular region of SURFIN1.1 of 31 field isolates compared to P. falciparum 3D7 reference strain. SURFIN1.1 sequences from 31 patients-isolated were employed in the analyses. Sequence analyses revealed identity and mutation amino acid residues were shown in each region.

**Additional file4: Figure S3.** Molecular phylogenetic analysis of SURFIN1.1 extracellular region from different haplotypes isolates in Thailand with the global reference strains. The evolution history was conducted in MEGA X using the Maximum Likelihood method based on the Hasegawa-Kishino-Yano model. Bootstrap values below 50 are not shown. The reference strains included 3D7, NF54, 7G8, HB3, Dd2, and D10.

## Data Availability

The data sets used in this study are available from the corresponding author on a reasonable request. All nucleotide sequences used and analysed in this study have been deposited at DDBJ/ENA/GenBank under the accession number MW767839-767869.

## Abbreviations

Aa: Amino acid
CRD: Cysteine-rich domain
ER: Endoplasmic reticulum
nt: Nucleotide
N-ter: N-terminal region
PfEMP-1: *P. falciparum* erythrocyte membrane protein-1
PEXEL: Plasmodium export element
PV: Parasitophorous vacuole
REX: Ring exported proteins
RIFINs: Repetitive interspersed family proteins
SUFRIN: Surface-associated interspersed protein
*surf*: Surface-associated interspersed gene
TM: Transmembrane
Var: Variable region
WRD: Tryptophan-rich domain
