# Supplementary material for: Genetic polymorphism of the extracellular region in surface associated interspersed 1.1 gene of Plasmodium falciparum field isolates from Thailand
Source: Malar J. 2021 Aug 16;20:343. doi: 10.1186/s12936-021-03876-y (PMC8365296; doi:10.1186/s12936-021-03876-y)
Supplement: Supplementary file 1 — Additional file 1: Table S1. Nucleotide identity among 31 field isolates (excepted for isolate A18) and P. falciparum 3D7 reference strain. Table S2. Amino acid identity among 31 field isolates (excepted for isolate A18) and P. falciparum 3D7 reference strain. [file 12936_2021_3876_MOESM1_ESM.docx]

**Table S1** Nucleotide identity among 31 field isolates (excepted for isolate A18) and *P. falciparum* 3D7 reference strain

| **% Identity** | **% Identity** | | | | | | | | | | | | | | | | | | | | | | | | | | | | | | | | |
| --- | --- | --- | --- | --- | --- | --- | --- | --- | --- | --- | --- | --- | --- | --- | --- | --- | --- | --- | --- | --- | --- | --- | --- | --- | --- | --- | --- | --- | --- | --- | --- | --- | --- |
|  |  | 3D7 | A1 | A2 | A3 | A4 | A5 | A6 | A7 | A8 | A9 | A10 | A11 | A12 | A13 | A14 | A15 | A16 | A17 | A19 | A20 | A21 | A22 | A23 | A24 | A25 | A26 | A27 | A28 | A29 | A30 | A31 | A32 |
|  | 3D7 |  | 99.3 | 99.2 | 99.4 | 99.4 | 99.2 | 99.2 | 99.4 | 99.4 | 99.2 | 99.2 | 99.2 | 99.2 | 99.2 | 99.4 | 99.4 | 99.4 | 99.2 | 99.2 | 99.2 | 99.2 | 99.2 | 99.4 | 99.4 | 99.2 | 99.2 | 99.4 | 99.2 | 99.2 | 99.2 | 98.9 | 99.2 |
|  | A1 | 99.3 |  | 99.0 | 99.4 | 99.0 | 99.0 | 99.0 | 99.4 | 99.4 | 99.0 | 99.0 | 99.0 | 99.0 | 99.0 | 99.4 | 99.9 | 99.4 | 99.0 | 99.0 | 99.0 | 99.0 | 99.0 | 99.9 | 99.9 | 99.0 | 99.0 | 99.9 | 99.0 | 99.0 | 99.0 | 98.7 | 99.0 |
|  | A2 | 99.2 | 99.0 |  | 99.6 | 99.6 | 100.0 | 100.0 | 99.6 | 99.6 | 100.0 | 100.0 | 100.0 | 100.0 | 100.0 | 99.6 | 99.1 | 99.6 | 100.0 | 100.0 | 100.0 | 100.0 | 100.0 | 99.1 | 99.1 | 100.0 | 100.0 | 99.1 | 100.0 | 100.0 | 100.0 | 99.4 | 100.0 |
|  | A3 | 99.4 | 99.4 | 99.6 |  | 99.6 | 99.6 | 99.6 | 100.0 | 100.0 | 99.6 | 99.6 | 99.6 | 99.6 | 99.6 | 100.0 | 99.6 | 100.0 | 99.6 | 99.6 | 99.6 | 99.6 | 99.6 | 99.6 | 99.6 | 99.6 | 99.6 | 99.6 | 99.6 | 99.6 | 99.6 | 99.2 | 99.6 |
|  | A4 | 99.4 | 99.0 | 99.6 | 99.6 |  | 99.6 | 99.6 | 99.6 | 99.6 | 99.6 | 99.6 | 99.6 | 99.6 | 99.6 | 99.6 | 99.1 | 99.6 | 99.6 | 99.6 | 99.6 | 99.6 | 99.6 | 99.1 | 99.1 | 99.6 | 99.6 | 99.1 | 99.6 | 99.6 | 99.6 | 99.0 | 99.6 |
|  | A5 | 99.2 | 99.0 | 100.0 | 99.6 | 99.6 |  | 100.0 | 99.6 | 99.6 | 100.0 | 100.0 | 100.0 | 100.0 | 100.0 | 99.6 | 99.1 | 99.6 | 100.0 | 100.0 | 100.0 | 100.0 | 100.0 | 99.1 | 99.1 | 100.0 | 100.0 | 99.1 | 100.0 | 100.0 | 100.0 | 99.4 | 100.0 |
|  | A6 | 99.2 | 99.0 | 100.0 | 99.6 | 99.6 | 100.0 |  | 99.6 | 99.6 | 100.0 | 100.0 | 100.0 | 100.0 | 100.0 | 99.6 | 99.1 | 99.6 | 100.0 | 100.0 | 100.0 | 100.0 | 100.0 | 99.1 | 99.1 | 100.0 | 100.0 | 99.1 | 100.0 | 100.0 | 100.0 | 99.4 | 100.0 |
|  | A7 | 99.4 | 99.4 | 99.6 | 100.0 | 99.6 | 99.6 | 99.6 |  | 100.0 | 99.6 | 99.6 | 99.6 | 99.6 | 100.0 | 100.0 | 99.6 | 100.0 | 99.6 | 99.6 | 99.6 | 99.6 | 99.6 | 99.6 | 99.6 | 99.6 | 99.6 | 99.6 | 99.6 | 99.6 | 99.6 | 99.2 | 99.6 |
|  | A8 | 99.4 | 99.4 | 99.6 | 100.0 | 99.6 | 99.6 | 99.6 | 100.0 |  | 99.6 | 99.6 | 99.6 | 99.6 | 99.6 | 100.0 | 99.6 | 100.0 | 99.6 | 99.6 | 99.6 | 99.6 | 99.6 | 99.6 | 99.6 | 99.6 | 99.6 | 99.6 | 99.6 | 99.6 | 99.6 | 99.2 | 99.6 |
|  | A9 | 99.2 | 99.0 | 100.0 | 99.6 | 99.6 | 100.0 | 100.0 | 99.6 | 99.6 |  | 100.0 | 100.0 | 100.0 | 100.0 | 99.6 | 99.1 | 99.6 | 100.0 | 100.0 | 100.0 | 100.0 | 100.0 | 99.1 | 99.1 | 100.0 | 100.0 | 99.1 | 100.0 | 100.0 | 100.0 | 99.4 | 100.0 |
|  | A10 | 99.2 | 99.0 | 100.0 | 99.6 | 99.6 | 100.0 | 100.0 | 99.6 | 99.6 | 100.0 |  | 100.0 | 100.0 | 100.0 | 99.6 | 99.1 | 99.6 | 100.0 | 100.0 | 100.0 | 100.0 | 100.0 | 99.1 | 99.1 | 100.0 | 100.0 | 99.1 | 100.0 | 100.0 | 100.0 | 99.4 | 100.0 |
|  | A11 | 99.2 | 99.0 | 100.0 | 99.6 | 99.6 | 100.0 | 100.0 | 99.6 | 99.6 | 100.0 | 100.0 |  | 100.0 | 100.0 | 99.6 | 99.1 | 99.6 | 100.0 | 100.0 | 100.0 | 100.0 | 100.0 | 99.1 | 99.1 | 100.0 | 100.0 | 99.1 | 100.0 | 100.0 | 100.0 | 99.4 | 100.0 |
|  | A12 | 99.2 | 99.0 | 100.0 | 99.6 | 99.6 | 100.0 | 100.0 | 99.6 | 99.6 | 100.0 | 100.0 | 100.0 |  | 100.0 | 99.6 | 99.1 | 99.6 | 100.0 | 100.0 | 100.0 | 100.0 | 100.0 | 99.1 | 99.1 | 100.0 | 100.0 | 99.1 | 100.0 | 100.0 | 100.0 | 99.4 | 100.0 |
|  | A13 | 99.2 | 99.0 | 100.0 | 99.6 | 99.6 | 100.0 | 100.0 | 100.0 | 99.6 | 100.0 | 100.0 | 100.0 | 100.0 |  | 99.6 | 99.1 | 99.6 | 100.0 | 100.0 | 100.0 | 100.0 | 100.0 | 99.1 | 99.1 | 100.0 | 100.0 | 99.1 | 100.0 | 100.0 | 100.0 | 99.4 | 100.0 |
|  | A14 | 99.4 | 99.4 | 99.6 | 100.0 | 99.6 | 99.6 | 99.6 | 100.0 | 100.0 | 99.6 | 99.6 | 99.6 | 99.6 | 99.6 |  | 99.6 | 100.0 | 99.6 | 99.6 | 99.6 | 99.6 | 99.6 | 99.6 | 99.6 | 99.6 | 99.6 | 99.6 | 99.6 | 99.6 | 99.6 | 99.2 | 99.6 |
|  | A15 | 99.4 | 99.9 | 99.1 | 99.6 | 99.1 | 99.1 | 99.1 | 99.6 | 99.6 | 99.1 | 99.1 | 99.1 | 99.1 | 99.1 | 99.6 |  | 99.6 | 99.1 | 99.1 | 99.1 | 99.1 | 99.1 | 100.0 | 100.0 | 99.1 | 99.1 | 100.0 | 99.1 | 99.1 | 99.1 | 98.8 | 99.1 |
|  | A16 | 99.4 | 99.4 | 99.6 | 100.0 | 99.6 | 99.6 | 99.6 | 100.0 | 100.0 | 99.6 | 99.6 | 99.6 | 99.6 | 99.6 | 100.0 | 99.6 |  | 99.6 | 99.6 | 99.6 | 99.6 | 99.6 | 99.6 | 99.6 | 99.6 | 99.6 | 99.6 | 99.6 | 99.6 | 99.6 | 99.2 | 99.6 |
|  | A17 | 99.2 | 99.0 | 100.0 | 99.6 | 99.6 | 100.0 | 100.0 | 99.6 | 99.6 | 100.0 | 100.0 | 100.0 | 100.0 | 100.0 | 99.6 | 99.1 | 99.6 |  | 100.0 | 100.0 | 100.0 | 100.0 | 99.1 | 99.1 | 100.0 | 100.0 | 99.1 | 100.0 | 100.0 | 100.0 | 99.4 | 100.0 |
|  | A19 | 99.2 | 99.0 | 100.0 | 99.6 | 99.6 | 100.0 | 100.0 | 99.6 | 99.6 | 100.0 | 100.0 | 100.0 | 100.0 | 100.0 | 99.6 | 99.1 | 99.6 | 100.0 |  | 100.0 | 100.0 | 100.0 | 99.1 | 99.1 | 100.0 | 100.0 | 99.1 | 100.0 | 100.0 | 100.0 | 99.4 | 100.0 |
|  | A20 | 99.2 | 99.0 | 100.0 | 99.6 | 99.6 | 100.0 | 100.0 | 99.6 | 99.6 | 100.0 | 100.0 | 100.0 | 100.0 | 100.0 | 99.6 | 99.1 | 99.6 | 100.0 | 100.0 |  | 100.0 | 100.0 | 99.1 | 99.1 | 100.0 | 100.0 | 99.1 | 100.0 | 100.0 | 100.0 | 99.4 | 100.0 |
|  | A21 | 99.2 | 99.0 | 100.0 | 99.6 | 99.6 | 100.0 | 100.0 | 99.6 | 99.6 | 100.0 | 100.0 | 100.0 | 100.0 | 100.0 | 99.6 | 99.1 | 99.6 | 100.0 | 100.0 | 100.0 |  | 100.0 | 99.1 | 99.1 | 100.0 | 100.0 | 99.1 | 100.0 | 100.0 | 100.0 | 99.4 | 100.0 |
|  | A22 | 99.2 | 99.0 | 100.0 | 99.6 | 99.6 | 100.0 | 100.0 | 99.6 | 99.6 | 100.0 | 100.0 | 100.0 | 100.0 | 100.0 | 99.6 | 99.1 | 99.6 | 100.0 | 100.0 | 100.0 | 100.0 |  | 99.1 | 99.1 | 100.0 | 100.0 | 99.1 | 100.0 | 100.0 | 100.0 | 99.4 | 100.0 |
|  | A23 | 99.4 | 99.9 | 99.1 | 99.6 | 99.1 | 99.1 | 99.1 | 99.6 | 99.6 | 99.1 | 99.1 | 99.1 | 99.1 | 99.1 | 99.6 | 100.0 | 99.6 | 99.1 | 99.1 | 99.1 | 99.1 | 99.1 |  | 100.0 | 99.1 | 99.1 | 100.0 | 99.1 | 99.1 | 99.1 | 98.8 | 99.1 |
|  | A24 | 99.4 | 99.9 | 99.1 | 99.6 | 99.1 | 99.1 | 99.1 | 99.6 | 99.6 | 99.1 | 99.1 | 99.1 | 99.1 | 99.1 | 99.6 | 100.0 | 99.6 | 99.1 | 99.1 | 99.1 | 99.1 | 99.1 | 100.0 |  | 99.1 | 99.1 | 100.0 | 99.1 | 99.1 | 99.1 | 98.8 | 99.1 |
|  | A25 | 99.2 | 99.0 | 100.0 | 99.6 | 99.6 | 100.0 | 100.0 | 99.6 | 99.6 | 100.0 | 100.0 | 100.0 | 100.0 | 100.0 | 99.6 | 99.1 | 99.6 | 100.0 | 100.0 | 100.0 | 100.0 | 100.0 | 99.1 | 99.1 |  | 100.0 | 99.1 | 100.0 | 100.0 | 100.0 | 99.4 | 100.0 |
|  | A26 | 99.2 | 99.0 | 100.0 | 99.6 | 99.6 | 100.0 | 100.0 | 99.6 | 99.6 | 100.0 | 100.0 | 100.0 | 100.0 | 100.0 | 99.6 | 99.1 | 99.6 | 100.0 | 100.0 | 100.0 | 100.0 | 100.0 | 99.1 | 99.1 | 100.0 |  | 99.1 | 100.0 | 100.0 | 100.0 | 99.4 | 100.0 |
|  | A27 | 99.4 | 99.9 | 99.1 | 99.6 | 99.1 | 99.1 | 99.1 | 99.6 | 99.6 | 99.1 | 99.1 | 99.1 | 99.1 | 99.1 | 99.6 | 100.0 | 99.6 | 99.1 | 99.1 | 99.1 | 99.1 | 99.1 | 100.0 | 100.0 | 99.1 | 99.1 |  | 99.1 | 99.1 | 99.1 | 98.8 | 99.1 |
|  | A28 | 99.2 | 99.0 | 100.0 | 99.6 | 99.6 | 100.0 | 99.6 | 99.6 | 99.6 | 100.0 | 100.0 | 100.0 | 100.0 | 100.0 | 99.6 | 99.1 | 99.6 | 100.0 | 100.0 | 100.0 | 100.0 | 100.0 | 99.1 | 99.1 | 100.0 | 100.0 | 99.1 |  | 100.0 | 100.0 | 99.4 | 100.0 |
|  | A29 | 99.2 | 99.0 | 100.0 | 99.6 | 99.6 | 100.0 | 99.6 | 99.6 | 99.6 | 100.0 | 100.0 | 100.0 | 100.0 | 100.0 | 99.6 | 99.1 | 99.6 | 100.0 | 100.0 | 100.0 | 100.0 | 100.0 | 99.1 | 99.1 | 100.0 | 100.0 | 99.1 | 100.0 |  | 100.0 | 99.4 | 100.0 |
|  | A30 | 99.2 | 99.0 | 100.0 | 99.6 | 99.6 | 100.0 | 99.6 | 99.6 | 99.6 | 100.0 | 100.0 | 100.0 | 100.0 | 100.0 | 99.6 | 99.1 | 99.6 | 100.0 | 100.0 | 100.0 | 100.0 | 100.0 | 99.1 | 99.1 | 100.0 | 100.0 | 99.1 | 100.0 | 100.0 |  | 99.4 | 100.0 |
|  | A31 | 98.9 | 98.7 | 99.4 | 99.2 | 99.0 | 99.4 | 99.4 | 99.2 | 99.2 | 99.4 | 99.4 | 99.4 | 99.4 | 99.4 | 99.2 | 98.8 | 99.2 | 99.4 | 99.4 | 99.4 | 99.4 | 99.4 | 98.8 | 98.8 | 99.4 | 99.4 | 98.8 | 99.4 | 99.4 | 99.4 |  | 99.4 |
|  | A32 | 99.2 | 99.0 | 100.0 | 99.6 | 99.6 | 100.0 | 100.0 | 99.6 | 99.6 | 100.0 | 100.0 | 100.0 | 100.0 | 100.0 | 99.6 | 99.1 | 99.6 | 100.0 | 100.0 | 100.0 | 100.0 | 100.0 | 99.1 | 99.1 | 100.0 | 100.0 | 99.1 | 100.0 | 100.0 | 100.0 | 99.4 |  |

**Table S2** Amino acid identity among 31 field isolates (excepted for isolate A18) and *P. falciparum* 3D7 reference strain

| **% Identity** | | | | | | | | | | | | | | | | | | | | | | | | | | | | | | | | | |
| --- | --- | --- | --- | --- | --- | --- | --- | --- | --- | --- | --- | --- | --- | --- | --- | --- | --- | --- | --- | --- | --- | --- | --- | --- | --- | --- | --- | --- | --- | --- | --- | --- | --- |
| **% Identity** |  | 3D7 | A1 | A2 | A3 | A4 | A5 | A6 | A7 | A8 | A9 | A10 | A11 | A12 | A13 | A14 | A15 | A16 | A17 | A19 | A20 | A21 | A22 | A23 | A24 | A25 | A26 | A27 | A28 | A29 | A30 | A31 | A32 |
|  | 3D7 |  | 98.3 | 98.0 | 98.7 | 98.3 | 98.0 | 98.0 | 98.7 | 98.7 | 98.0 | 98.0 | 98.0 | 98.0 | 98.0 | 98.7 | 98.7 | 98.7 | 98.0 | 98.0 | 98.0 | 98.0 | 98.0 | 98.7 | 98.7 | 98.0 | 98.0 | 98.7 | 98.0 | 98.0 | 98.0 | 98.0 | 98.0 |
|  | A1 | 98.3 |  | 97.0 | 98.3 | 97.3 | 97.0 | 97.0 | 98.3 | 98.3 | 97.0 | 97.0 | 97.0 | 97.0 | 97.0 | 98.3 | 99.7 | 98.3 | 97.0 | 97.0 | 97.0 | 97.0 | 97.0 | 99.7 | 99.7 | 97.0 | 97.0 | 99.7 | 97.0 | 97.0 | 97.0 | 97.0 | 97.0 |
|  | A2 | 98.0 | 97.0 |  | 98.7 | 99.0 | 100.0 | 100.0 | 98.7 | 98.7 | 100.0 | 100.0 | 100.0 | 100.0 | 100.0 | 98.7 | 97.3 | 98.7 | 100.0 | 100.0 | 100.0 | 100.0 | 100.0 | 97.3 | 97.3 | 100.0 | 100.0 | 97.3 | 100.0 | 100.0 | 100.0 | 99.3 | 100.0 |
|  | A3 | 98.7 | 98.3 | 98.7 |  | 99.0 | 98.7 | 98.7 | 100.0 | 100.0 | 98.7 | 98.7 | 98.7 | 98.7 | 98.7 | 100.0 | 98.7 | 100.0 | 98.7 | 98.7 | 98.7 | 98.7 | 98.7 | 98.7 | 98.7 | 98.7 | 98.7 | 98.7 | 98.7 | 98.7 | 98.7 | 98.7 | 98.7 |
|  | A4 | 98.3 | 97.3 | 99.0 | 99.0 |  | 99.0 | 99.0 | 99.0 | 99.0 | 99.0 | 99.0 | 99.0 | 99.0 | 99.0 | 99.0 | 97.7 | 99.0 | 99.0 | 99.0 | 99.0 | 99.0 | 99.0 | 97.7 | 97.7 | 99.0 | 99.0 | 97.7 | 99.0 | 99.0 | 99.0 | 98.3 | 99.0 |
|  | A5 | 98.0 | 97.0 | 100.0 | 98.7 | 99.0 |  | 100.0 | 98.7 | 98.7 | 100.0 | 100.0 | 100.0 | 100.0 | 100.0 | 98.7 | 97.3 | 98.7 | 100.0 | 100.0 | 100.0 | 100.0 | 100.0 | 97.3 | 97.3 | 100.0 | 100.0 | 97.3 | 100.0 | 100.0 | 100.0 | 99.3 | 100.0 |
|  | A6 | 98.0 | 97.0 | 100.0 | 98.7 | 99.0 | 100.0 |  | 98.7 | 98.7 | 100.0 | 100.0 | 100.0 | 100.0 | 100.0 | 98.7 | 97.3 | 98.7 | 100.0 | 100.0 | 100.0 | 100.0 | 100.0 | 97.3 | 97.3 | 100.0 | 100.0 | 97.3 | 100.0 | 100.0 | 100.0 | 99.3 | 100.0 |
|  | A7 | 98.7 | 98.3 | 98.7 | 100.0 | 99.0 | 98.7 | 98.7 |  | 100.0 | 98.7 | 98.7 | 98.7 | 98.7 | 98.7 | 100.0 | 98.7 | 100.0 | 98.7 | 98.7 | 98.7 | 98.7 | 98.7 | 98.7 | 98.7 | 98.7 | 98.7 | 98.7 | 98.7 | 98.7 | 98.7 | 98.7 | 98.7 |
|  | A8 | 98.7 | 98.3 | 98.7 | 100.0 | 99.0 | 98.7 | 98.7 | 100.0 |  | 98.7 | 98.7 | 98.7 | 98.7 | 98.7 | 100.0 | 98.7 | 100.0 | 98.7 | 98.7 | 98.7 | 98.7 | 98.7 | 98.7 | 98.7 | 98.7 | 98.7 | 98.7 | 98.7 | 98.7 | 98.7 | 98.7 | 98.7 |
|  | A9 | 98.0 | 97.0 | 100.0 | 98.7 | 99.0 | 100.0 | 100.0 | 98.7 | 98.7 |  | 100.0 | 100.0 | 100.0 | 100.0 | 98.7 | 97.3 | 98.7 | 100.0 | 100.0 | 100.0 | 100.0 | 100.0 | 97.3 | 97.3 | 100.0 | 100.0 | 97.3 | 100.0 | 100.0 | 100.0 | 99.3 | 100.0 |
|  | A10 | 98.0 | 97.0 | 100.0 | 98.7 | 99.0 | 100.0 | 100.0 | 98.7 | 98.7 | 100.0 |  | 100.0 | 100.0 | 100.0 | 98.7 | 97.3 | 98.7 | 100.0 | 100.0 | 100.0 | 100.0 | 100.0 | 97.3 | 97.3 | 100.0 | 100.0 | 97.3 | 100.0 | 100.0 | 100.0 | 99.3 | 100.0 |
|  | A11 | 98.0 | 97.0 | 100.0 | 98.7 | 99.0 | 100.0 | 100.0 | 98.7 | 98.7 | 100.0 | 100.0 |  | 100.0 | 100.0 | 98.7 | 97.3 | 98.7 | 100.0 | 100.0 | 100.0 | 100.0 | 100.0 | 97.3 | 97.3 | 100.0 | 100.0 | 97.3 | 100.0 | 100.0 | 100.0 | 99.3 | 100.0 |
|  | A12 | 98.0 | 97.0 | 100.0 | 98.7 | 99.0 | 100.0 | 100.0 | 98.7 | 98.7 | 100.0 | 100.0 | 100.0 |  | 100.0 | 98.7 | 97.3 | 98.7 | 100.0 | 100.0 | 100.0 | 100.0 | 100.0 | 97.3 | 97.3 | 100.0 | 100.0 | 97.3 | 100.0 | 100.0 | 100.0 | 99.3 | 100.0 |
|  | A13 | 98.0 | 97.0 | 100.0 | 98.7 | 99.0 | 100.0 | 100.0 | 98.7 | 98.7 | 100.0 | 100.0 | 100.0 | 100.0 |  | 98.7 | 97.3 | 98.7 | 100.0 | 100.0 | 100.0 | 100.0 | 100.0 | 97.3 | 97.3 | 100.0 | 100.0 | 97.3 | 100.0 | 100.0 | 100.0 | 99.3 | 100.0 |
|  | A14 | 98.7 | 98.3 | 98.7 | 100.0 | 99.0 | 98.7 | 98.7 | 100.0 | 100.0 | 98.7 | 98.7 | 98.7 | 98.7 | 98.7 |  | 98.7 | 100.0 | 98.7 | 98.7 | 98.7 | 98.7 | 98.7 | 98.7 | 98.7 | 98.7 | 98.7 | 98.7 | 98.7 | 98.7 | 98.7 | 98.7 | 98.7 |
|  | A15 | 98.7 | 99.7 | 97.3 | 98.7 | 97.7 | 97.3 | 97.3 | 98.7 | 98.7 | 97.3 | 97.3 | 97.3 | 97.3 | 97.3 | 98.7 |  | 98.7 | 97.3 | 97.3 | 97.3 | 97.3 | 97.3 | 100.0 | 100.0 | 97.3 | 97.3 | 100.0 | 97.3 | 97.3 | 97.3 | 97.3 | 97.3 |
|  | A16 | 98.7 | 98.3 | 98.7 | 100.0 | 99.0 | 98.7 | 98.7 | 100.0 | 100.0 | 98.7 | 98.7 | 98.7 | 98.7 | 98.7 | 100.0 | 98.7 |  | 98.7 | 98.7 | 98.7 | 98.7 | 98.7 | 98.7 | 98.7 | 98.7 | 98.7 | 98.7 | 98.7 | 98.7 | 98.7 | 98.7 | 98.7 |
|  | A17 | 98.0 | 97.0 | 100.0 | 98.7 | 99.0 | 100.0 | 100.0 | 98.7 | 98.7 | 100.0 | 100.0 | 100.0 | 100.0 | 100.0 | 98.7 | 97.3 | 98.7 |  | 100.0 | 100.0 | 100.0 | 100.0 | 97.3 | 97.3 | 100.0 | 100.0 | 97.3 | 100.0 | 100.0 | 100.0 | 99.3 | 100.0 |
|  | A19 | 98.0 | 97.0 | 100.0 | 98.7 | 99.0 | 100.0 | 100.0 | 98.7 | 98.7 | 100.0 | 100.0 | 100.0 | 100.0 | 100.0 | 98.7 | 97.3 | 98.7 | 100.0 |  | 100.0 | 100.0 | 100.0 | 97.3 | 97.3 | 100.0 | 100.0 | 97.3 | 100.0 | 100.0 | 100.0 | 99.3 | 100.0 |
|  | A20 | 98.0 | 97.0 | 100.0 | 98.7 | 99.0 | 100.0 | 100.0 | 98.7 | 98.7 | 100.0 | 100.0 | 100.0 | 100.0 | 100.0 | 98.7 | 97.3 | 98.7 | 100.0 | 100.0 |  | 100.0 | 100.0 | 97.3 | 97.3 | 100.0 | 100.0 | 97.3 | 100.0 | 100.0 | 100.0 | 99.3 | 100.0 |
|  | A21 | 98.0 | 97.0 | 100.0 | 98.7 | 99.0 | 100.0 | 100.0 | 98.7 | 98.7 | 100.0 | 100.0 | 100.0 | 100.0 | 100.0 | 98.7 | 97.3 | 98.7 | 100.0 | 100.0 | 100.0 |  | 100.0 | 97.3 | 97.3 | 100.0 | 100.0 | 97.3 | 100.0 | 100.0 | 100.0 | 99.3 | 100.0 |
|  | A22 | 98.0 | 97.0 | 100.0 | 98.7 | 99.0 | 100.0 | 100.0 | 98.7 | 98.7 | 100.0 | 100.0 | 100.0 | 100.0 | 100.0 | 98.7 | 97.3 | 98.7 | 100.0 | 100.0 | 100.0 | 100.0 |  | 97.3 | 97.3 | 100.0 | 100.0 | 97.3 | 100.0 | 100.0 | 100.0 | 99.3 | 100.0 |
|  | A23 | 98.7 | 99.7 | 97.3 | 98.7 | 97.7 | 97.3 | 97.3 | 98.7 | 98.7 | 97.3 | 97.3 | 97.3 | 97.3 | 97.3 | 98.7 | 100.0 | 98.7 | 97.3 | 97.3 | 97.3 | 97.3 | 97.3 |  | 100.0 | 97.3 | 97.3 | 100.0 | 97.3 | 97.3 | 97.3 | 97.3 | 97.3 |
|  | A24 | 98.7 | 99.7 | 97.3 | 98.7 | 97.7 | 97.3 | 97.3 | 98.7 | 98.7 | 97.3 | 97.3 | 97.3 | 97.3 | 97.3 | 98.7 | 100.0 | 98.7 | 97.3 | 97.3 | 97.3 | 97.3 | 97.3 | 100.0 |  | 97.3 | 97.3 | 100.0 | 97.3 | 97.3 | 97.3 | 97.3 | 97.3 |
|  | A25 | 98.0 | 97.0 | 100.0 | 98.7 | 99.0 | 100.0 | 100.0 | 98.7 | 98.7 | 100.0 | 100.0 | 100.0 | 100.0 | 100.0 | 98.7 | 97.3 | 98.7 | 100.0 | 100.0 | 100.0 | 100.0 | 100.0 | 97.3 | 97.3 |  | 100.0 | 97.3 | 100.0 | 100.0 | 100.0 | 99.3 | 100.0 |
|  | A26 | 98.0 | 97.0 | 100.0 | 98.7 | 99.0 | 100.0 | 100.0 | 98.7 | 98.7 | 100.0 | 100.0 | 100.0 | 100.0 | 100.0 | 98.7 | 97.3 | 98.7 | 100.0 | 100.0 | 100.0 | 100.0 | 100.0 | 97.3 | 97.3 | 100.0 |  | 97.3 | 100.0 | 100.0 | 100.0 | 99.3 | 100.0 |
|  | A27 | 98.7 | 99.7 | 97.3 | 98.7 | 97.7 | 97.3 | 97.3 | 98.7 | 98.7 | 97.3 | 97.3 | 97.3 | 97.3 | 97.3 | 98.7 | 100.0 | 98.7 | 97.3 | 97.3 | 97.3 | 97.3 | 97.3 | 100.0 | 100.0 | 97.3 | 97.3 |  | 97.3 | 97.3 | 97.3 | 97.3 | 97.3 |
|  | A28 | 98.0 | 97.0 | 100.0 | 98.7 | 99.0 | 100.0 | 100.0 | 98.7 | 98.7 | 100.0 | 100.0 | 100.0 | 100.0 | 100.0 | 98.7 | 97.3 | 98.7 | 100.0 | 100.0 | 100.0 | 100.0 | 100.0 | 97.3 | 97.3 | 100.0 | 100.0 | 97.3 |  | 100.0 | 100.0 | 99.3 | 100.0 |
|  | A29 | 98.0 | 97.0 | 100.0 | 98.7 | 99.0 | 100.0 | 100.0 | 98.7 | 98.7 | 100.0 | 100.0 | 100.0 | 100.0 | 100.0 | 98.7 | 97.3 | 98.7 | 100.0 | 100.0 | 100.0 | 100.0 | 100.0 | 97.3 | 97.3 | 100.0 | 100.0 | 97.3 | 100.0 |  | 100.0 | 99.3 | 100.0 |
|  | A30 | 98.0 | 97.0 | 100.0 | 98.7 | 99.0 | 100.0 | 100.0 | 98.7 | 98.7 | 100.0 | 100.0 | 100.0 | 100.0 | 100.0 | 98.7 | 97.3 | 98.7 | 100.0 | 100.0 | 100.0 | 100.0 | 100.0 | 97.3 | 97.3 | 100.0 | 100.0 | 97.3 | 100.0 | 100.0 |  | 99.3 | 100.0 |
|  | A31 | 98.0 | 97.0 | 99.3 | 98.7 | 98.3 | 99.3 | 99.3 | 98.7 | 98.7 | 99.3 | 99.3 | 99.3 | 99.3 | 99.3 | 98.7 | 97.3 | 98.7 | 99.3 | 99.3 | 99.3 | 99.3 | 99.3 | 97.3 | 97.3 | 99.3 | 99.3 | 97.3 | 99.3 | 99.3 | 99.3 |  | 99.3 |
|  | A32 | 98.0 | 97.0 | 100.0 | 98.7 | 99.0 | 100.0 | 100.0 | 98.7 | 98.7 | 100.0 | 100.0 | 100.0 | 100.0 | 100.0 | 98.7 | 97.3 | 98.7 | 100.0 | 100.0 | 100.0 | 100.0 | 100.0 | 97.3 | 97.3 | 100.0 | 100.0 | 97.3 | 100.0 | 100.0 | 100.0 | 99.3 |  |
