# Supplementary figures and images for: Genetic polymorphism of the extracellular region in surface associated interspersed 1.1 gene of Plasmodium falciparum field isolates from Thailand
Source: Malar J. 2021 Aug 16;20:343. doi: 10.1186/s12936-021-03876-y (PMC8365296; doi:10.1186/s12936-021-03876-y)

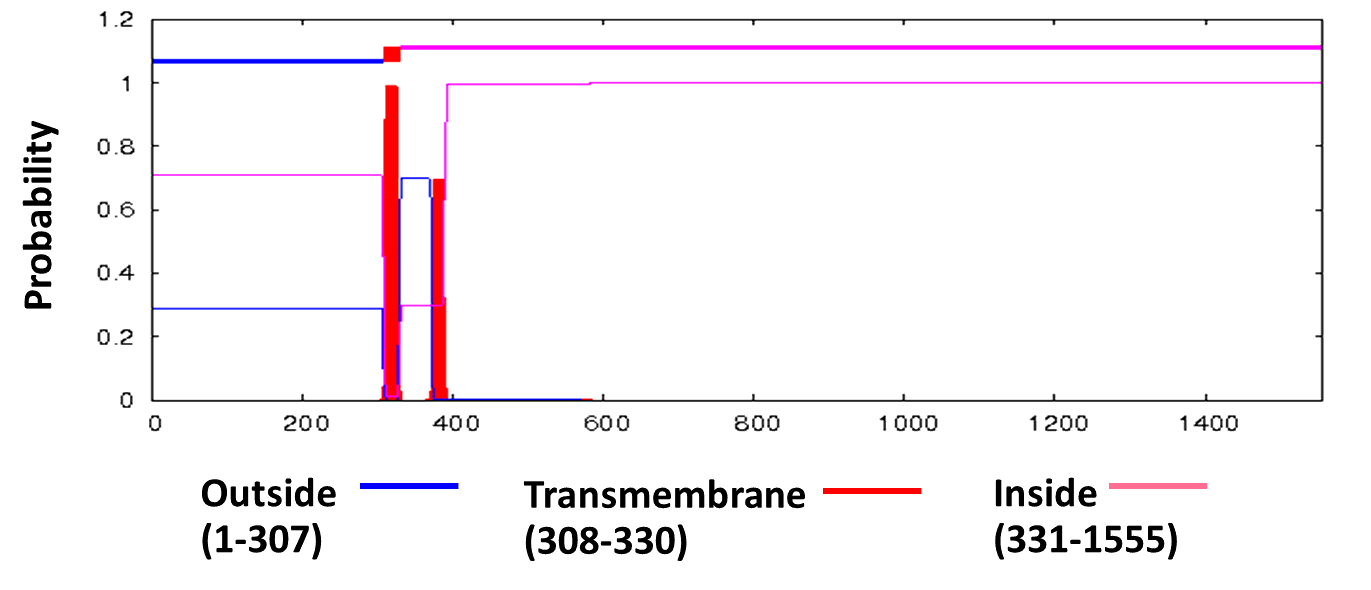

Supplement: Supplementary file 2 — Additional file2: Figure S1. The predicted transmembrane region (TM) of SURFIN1.1. The TM was predicted by using TMHMM servers. The predicted TM of SURFIN1.1 was located at amino acid residues 308 to 330. [file 12936_2021_3876_MOESM2_ESM.png]

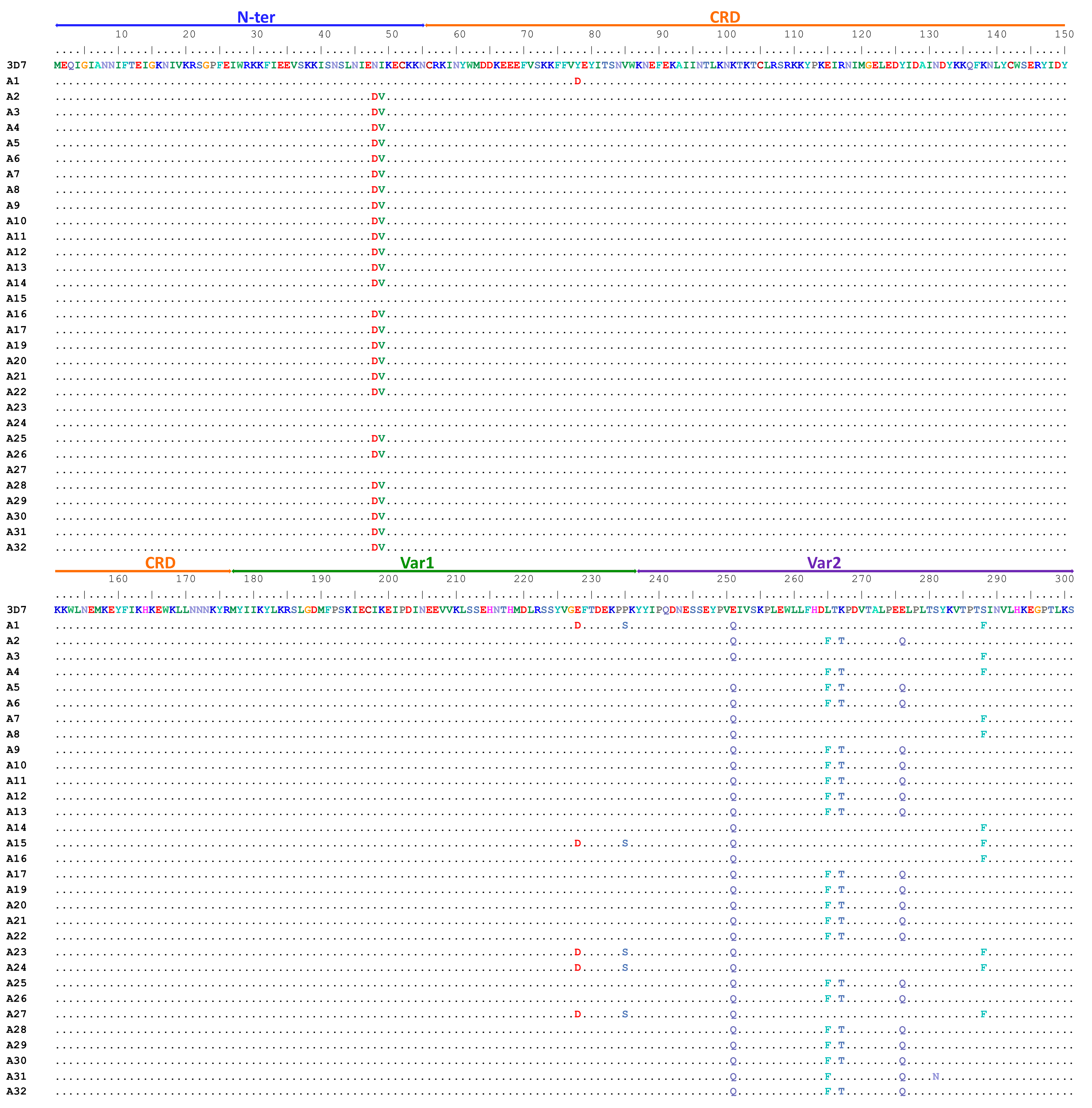

Supplement: Supplementary file 3 — Additional file3: Figure S2. Amino acid sequences alignment of the extracellular region of SURFIN1.1 of 31 field isolates compared to P. falciparum 3D7 reference strain. SURFIN1.1 sequences from 31 patients-isolated were employed in the analyses. Sequence analyses revealed identity and mutation amino acid residues were shown in each region. [file 12936_2021_3876_MOESM3_ESM.png]

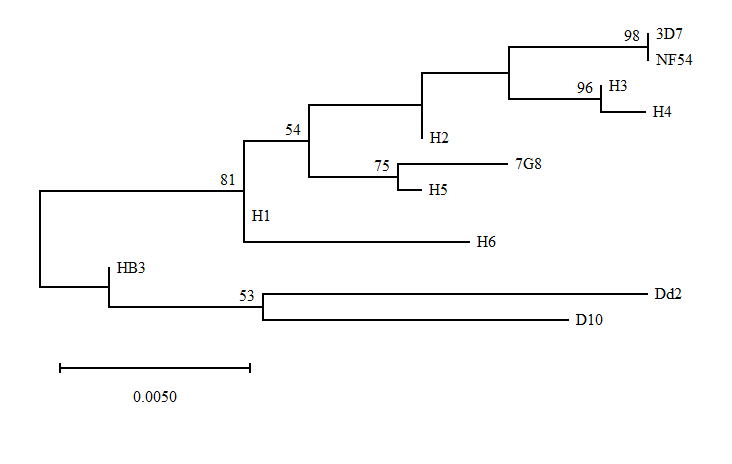

Supplement: Supplementary file 4 — Additional file4: Figure S3. Molecular phylogenetic analysis of SURFIN1.1 extracellular region from different haplotypes isolates in Thailand with the global reference strains. The evolution history was conducted in MEGA X using the Maximum Likelihood method based on the Hasegawa-Kishino-Yano model. Bootstrap values below 50 are not shown. The reference strains included 3D7, NF54, 7G8, HB3, Dd2, and D10. [file 12936_2021_3876_MOESM4_ESM.png]
